# Vision Loss and Psychological Distress among Ethiopians Adults: A Comparative Cross-Sectional Study

**DOI:** 10.1371/journal.pone.0078335

**Published:** 2013-10-25

**Authors:** Aemero Abateneh, Markos Tesfaye, Sisay Bekele, Yeshigeta Gelaw

**Affiliations:** 1 Department of Ophthalmology, College of Public Health and Medical Sciences, Jimma University, Jimma, Ethiopia; 2 Department of Psychiatry, College of Public Health and Medical Sciences, Jimma University, Jimma, Ethiopia; Medical University Graz, Austria

## Abstract

**Background:**

Vision loss causes major changes in lifestyle and habits that may result in psychological distress and further reduction in the quality of life. Little is known about the magnitude of psychological distress in patients with vision loss and its variation with the normal. The aim of this study is, therefore, to investigate the psychological effects of vision loss and its determinants among Ethiopians.

**Methods:**

A comparative cross-sectional study was conducted on adults attending the Eye clinic of Jimma University Hospital. One hundred fifteen consecutive adults with visual loss at least in one eye and 115 age-and sex-matched controls with normal vision were studied. The psychological distress was measured using standardized Self-Reporting Questionnaire (SRQ-20). Chi-square test and logistic regression were carried out and associations were considered significant at *P*<0.05.

**Results:**

The overall prevalence of psychological distress was 33.4%. While psychological distress was found in 49.8% of patients who had loss of vision at least in one eye, only 18.3% of the controls had it. In the adjusted analysis, patients with vision loss had 4.6 times higher risk of suffering from psychological distress compared to patients with normal vision (AOR 4.56; 95% CI 2.16-9.62). Moreover, patients with vision loss in both eyes (AOR 4.00; 95% CI 1.453-11.015) and with worse visual acuity in the better eye (AOR 3.66; 95% CI 1.27-10.54) were significantly more likely to have psychological distress than those patients with vision loss in one eye only and good visual acuity in the better eye respectively. The cause of visual loss, pattern of visual loss, duration of visual loss and sociodemographic variables did not influence the likelihood of having psychological distress.

**Conclusion:**

Prevalence of psychological distress was significantly higher in patients with visual loss compared to patients with normal vision. There is a need for integration of psychosocial care into the current medical and surgical treatment of patients with vision loss.

## Introduction

Eyes play a key role in all life forms to perceive and react to their environment. About 85-90% of the sensory input to the brain comes from visual perception making vision critical and essential for a human being to be connected to the world [1-3]. Hence, any significant loss of visual function will diminish our ability to survive on the planet earth as we will be unable to fully sense danger and lose a great deal of interpersonal communication, education and social world [1-3] and other various independent living skills like mobility, which variously results in a loss of personal independence, social and family role, psychological and financial security, and ultimately self-esteem [2,3].

When a person loses his/her sight and becomes blind, a devastating condition with deep psychological and socioeconomic implications follows. These consequences affect not only the person but also the family and the community. The loss of vision either from eye diseases or trauma causes major changes in lifestyle and habits of the person [4]. These changes may result in psychosocial distress leading to maladjustment [5] and further reduction of one’s quality of life [5]. Various studies revealed that visual impairment causes lower level of psychological health, social functioning and profoundly reduced quality of life [6-10]. The potential risk factors for emotional distress among visually impaired or blind patients include shorter duration of ocular disease, worse visual acuity, presence of other co-morbid systemic illnesses and functional impairment, poor educational background, lower socioeconomic class, rural residence, illiteracy and unmarried life [9-11]. One of the few existing published literatures by Dupe S et al from Nigeria showed that 51% of patients with bilateral visual loss had significant psychological distress (SRQ ≥ 5) [12]. 

Low vision and blindness are recognized as one of the major public health problems worldwide, especially in developing countries. According to WHO 2010 estimate, there are 285 million visually impaired people worldwide, and of these 39 million are blind [13]. However, the health care focus for these blind and visually impaired people is on the medical and surgical ophthalmic care. The psychological aspect is often ignored or it is of less priority to the ophthalmic community. This has led to the provision of highly specialized care to the eye but partial care to the patient in totality. The lack of enough literature on visual loss and psychological distress has contributed to the negligence of the psychological care of patients with visual loss or blindness. Given the potentially harmful effect of co-morbid psychological distress on the functioning and quality of life of patients with vision loss, there is an urgent need for research in the area. Generating evidence on the prevalence and severity of psychological distress among patients with visual loss and its associated risk factors will help to attract more attention and improve the clinical decision-making and the psychosocial care of such patients. This study thus aims to investigate the association between loss of vision and psychological distress among adult outpatients attending the Eye Clinic in rural Southwest Ethiopia.

## Methods

### Ethics Statement

We carried out research following the tenets of Declaration of Helsinki, and the Institutional Review Board of Jimma University approved the study. We explained the nature and the objective of the study and possible consequences of the study using their local language and the study participants and/or the care takers signed informed consent. We ensured that the psychological distress measures of the participants were entirely anonymous, and we did not record any identifying information on our data collection format. All patients received psychological counseling, medical and surgical treatment as needed**.**

### Study Design, Setting, and Participant

We conducted a comparative cross-sectional study at Jimma University Teaching Hospital (JUTH), Department of Ophthalmology, Southwest Ethiopia in 2012. The hospital is the only referral center in the region serving to a population of about 15 millions. We recruited a consecutive sample of patients older than 15 years of age and with Snellen visual acuity of < 3/60 at least in one eye. Age- and sex-matched controls, without visual impairment, attending the eye clinic during the study period were also took part in the study. Patients who had severe cognitive or psychiatric conditions that could interfere with communication were excluded.

We estimated the sample size of persons with visual loss (exposed) and persons with normal vision (unexposed/controls) using the Fleiss corrected sample size calculation formula (Epi Info ^TM^). As the prevalence of psychological distress in Ethiopian community (unexposed) is 17% [14], with risk ratio of 2, power of 80%, and 1:1 ratio of exposed and unexposed; the sample size in each group became 114 at 95% confidence interval.

### Data collection and measurements

We collected the data using interviews and clinical examination techniques. The interview had two sections: background information and psychological distress measure. We used structured questionnaire to ask about socioeconomic and demographic characteristics; and presence of co-morbid medical illness. We measured the psychological distress by standardized WHO self-reporting questionnaire (SRQ-20) [15]. The SRQ-20 has been adapted, validated and extensively used in the Ethiopian setting to measure psychological morbidity both in the community and general medical settings [14,16-19]. The SRQ-20 has 20 questions asking whether the person had experienced specific symptoms over the previous one month period. Those patients who gave 11 or more positive responses to SRQ-20 were regarded as having psychological distress as suggested by other studies [14,17]. We also reviewed patients’ ophthalmic record/chart to determine the cause of their visual loss and categorize visual impairment according to the WHO definition [20]. In circumstances where the chart was incomplete, we reassessed visual acuity with Snellen acuity chart and did standard anterior and posterior segment examinations using Slit Lamp Biomicroscope and 90 Diopter Volk lens.

### Covariates

Covariates in our analysis included age, sex, marital status, religion, level of education, occupation, family monthly income, visual status (loss of vision at least in one eye/normal vision), residence (urban/rural), family size, living arrangement, having other co-morbid medical illness (yes/no), SRQ score (>11/<11), level of visual acuity in the better eye (>3/60/<3/60), laterality of visual loss (one eye/both eyes), pattern of visual loss (progressive/sudden), cause of visual loss, and duration of visual loss (<1 year/>1 year).

### Data analysis

We analyzed the data using SPSS version 16 statistical software (SPSS Inc., Chicago, IL, USA). We performed Chi-square tests to check for statistically significant differences between the study groups and control groups in terms of various characteristics. We did univariate regression analysis to find potential predictors of psychological distress, and multiple logistic regressions to control for confounding and compute Adjusted Odds Ratios [AOR] with 95% confidence interval. We considered the statistical tests significant if *P* < 0.05. 

## Results

Of the 230 study subjects, 115 were patients with vision loss at least in one eye and 115 were age-and sex-matched patients with normal vision. Majority of the patients were 45 years old and above (62.6%), males (75.7%), married (63.5%), and Orthodox Christians (47%). About 62% of the participants had attained elementary school and above, and most of them (68.6%) had an estimated average monthly family income of less than 1000 Birr (≈60 USD) (Table 1). The mean age for patients with vision loss (cases) and normal vision (controls) was 48.15 (SD+17) and 48.10 (SD+17.2) years respectively with no statistically significant age difference in the two groups (*P*=0.118). The sex distribution was also equal in both groups (*P*=0.561). However, patients in the two groups differed significantly in place of residence (*P*=0.000), religion (*P*=0.003), level of education (*P*=0.0001), occupation (*P*=0.001) and family income (*P*=0.005) (Table 1). 

**Table 1:** Sociodemographic characteristics of the study subjects.

**Variables**	**Population**		**Total**	**X^2^**	**P - value**
	**Loss of vision**	**Normal vision**			
	**No (%)**	**No (%)**	**No (%)**		
**Age**				6.418	0.118
15-24	14(12.2)	14( 12.2)	28(12.1)		
25-44	28(24.3)	30(26.1)	58(25.2)		
45-60	42(36.5)	41(35.7)	83(36.1)		
60+	31(27)	30(26.1)	61(26.5)		
**Sex**				0.000	0.561
Male	87(75.7)	87(75.7)	174(75.7)		
Female	28(24.3)	28(24.3)	56(24.3)		
**Marital status**				0.838	0.840
Single	25(10.9)	26(11.3)	51(22.2)		
Married	74(32.2)	72(33.3)	146(63.5)		
Separated/divorced	10(4.3)	13(5.7)	23(10.0)		
Widowed	6(2.6)	4(1.7)	10(4.3)		
**Residence**				30.810	0.000
Rural	65(56.5)	24(20.9)	89(38.7)		
Urban	50(43.5)	91(79.1)	141(61.3)		
**Family size**				6.418	0.093
1-2	16(39.0)	25(61.0)	41(17.8)		
3-5	51(60.0)	34(40.0)	85(37.0)		
6-8	37(48.1)	40(51.9)	77(33.5)		
9+	11(40.7)	16(59.3)	27(11.7)		
**Level of education**				29.430	0.0001
Illiterate	43(37.4)	19(16.5)	62(27.0)		
Read and write	17(14.8)	9(7.8)	26(11.3)		
Elementary school	37(32.2)	34(29.6)	71(30.9)		
Secondary school	9(7.8)	22(19.1)	31(13.5)		
College and above	9(7.8)	31(27.0)	40(17.4)		
**Occupation**				23.607	0.001
Farmer	55(47.8)	27(23.5)	82(35.7)		
Skilled laborer	13(11.3)	30(26.1)	43(18.7)		
Unskilled laborer	16(13.9)	19(16.5)	35(15.2)		
Student	6(5.2)	10(8.7)	16(7.0)		
House wife	11(9.5)	5(4.3)	16(7.0)		
Own business	8(7.0)	10(8.7)	18(7.8)		
Retired	4(3.5)	7(6.1)	11(4.8)		
Jobless	2(1.7)	7(6.1)	9(3.9)		
**Religion**				13.827	0.003
Muslim	60(52.2)	34(29.6)	94(40.9)		
Orthodox Christian	43(37.4)	65(56.5)	108(47)		
Protestant	12(10.4)	14(12.2)	26(11.3)		
Others	0(0.0)	2(1.7)	2(0.9)		
**Living arrangement**				2.210	0.530
Alone	12(10.4)	17(14.8)	29(12.6)		
With family	96(83.5)	88(76.5)	184(80.0)		
With other relatives	4(3.5)	4(3.5)	8(3.5)		
Others	3(2.6)	6(5.2)	9(3.9)		
**Family income per month in birr**				15.025	0.005
<500	45(39.1)	34(29.6)	79(34.3)		
500-1000	45(39.1)	34(29.6)	79(34.3)		
1000-1500	14(12.2)	15(13.0)	29(12.6)		
1500-2000	8(7.0)	15(13.0)	23(10.0)		
>2000	3(2.6)	17(14.8)	20(8.7)		
**Having other medical illness**				1.310	0.2520
Yes	20(17.4)	27(23.5)	47(20.4)		
No	95(82.6)	88(76.5)	183(79.6)		
**SRQ score**					
<11	58(50.4)	94(81.7)	152(66.1)	21.149	0.000
>11	57(49.6)	21(18.3)	78(33.9)		

The overall prevalence of psychological distress in the study population was 33.9%, and the prevalence was higher (49.6%) among people with visual loss than patients with normal vision 18.3% (Table 1). In bivariate analysis marital status, visual acuity, occupation, level of education, family income and living arrangement were significantly associated with psychological distress (*P* < 0.05). However, age, sex, religion, residency, family size and having other co-morbid illness were not significantly associated with psychological distress (*P* > 0.05). In multivariate logistic regression analysis, psychological distress was significantly associated with vision loss (*P* <0.05). After controlling for other sociodemographic variables, the likelihood of having psychological distress was 5-fold higher among patients who lost vision at least in one eye compared to patients who had normal vision (AOR 4.5; 95% CI 2.161- 9.617). The effect of marital status, educational status, occupational status, family income and living arrangement on psychological distress failed to persist after controlling for other demographic factors (Table 2). 

**Table 2:** Crude and adjusted ratios of psychological distress, demographic characteristics and level of visual acuity.

**Variables**	**Total**	**Cases**	**COR**	**95 % CI**	**AOR**	**95 % CI**
	**No**	**No (%)**				
**Marital status**						
Single	51	11(21.6)	1		1	
Married	146	47(32.2)	1.726	0.814, 3.663	1.503	0.473,4.776
Separated/divorced	23	12(52.2)	3.967	1.380, 11.401	2.094	0.529,8.292
Widowed	10	8(80.0)	14.54	2.692,78.597	6.176	0.835,45.666
**Level of education**						
College and above	40	8(20.0)	1		1	
Secondary	31	6(19.4)	0.960	1.931,12.215	0.735	0.134,4.028
Elementary	71	17(23.9)	1.259	1.343, 11.914	0.510	0.080,3.242
Read and write	26	13(50.0)	4.000	0.488, 3.247	1.064	0.139,8.129
Illiterate	62	34(54.8)	4.857	0.295, 3.126	1.131	0.154,8.291
**Occupation**						
Jobless	9	5(55.6)	1		1	
Retired	11	4(36.4)	0.457	0.076, 2.764	1.527	0.141,16.567
Own business	18	3(16.7)	0.160	0.026,0.975	0.712	0.065,7.754
House wife	16	6(37.5)	0.480	0.091, 2.523	0.641	0.064,6.410
Student	16	0(0.0)	0.000	0.000	0.000	0.000
Unskilled laborer	35	14(40.0)	0.533	0.122,2.339	1.161	0.139,9.692
Skilled laborer	43	9(20.9)	0.212	0.047,0.955	0.814	0.057,11.706
Farmer	82	37(45.1)	0.658	0.165, 2.627	1.322	0.178,9.818
**Living arrangements**						
Alone	29	12(41.4)	1		1	
With family	184	59(32.1)	0.669	0.300, 1.490	0.457	0.121,1.729
With other relatives	8	6(75.0)	4.250	0.729, 24.769	2.744	0.168,44.808
Others	9	1(11.1)	0.177	0.019, 1.608	1.919	0.000
**Family income per month in birr**						
>2000	20	1(5.0)	1		1	
1500-2000	23	6(26.1)	6.706	2.364, 45.155	4.082	0.410,40.619
1000 -1500	29	8(27.6)	7.238	1.049, 65.524	3.547	0.357,35.194
500-1000	79	24(30.4)	8.291	0.827, 63.362	3.420	0.366,31.964
<500	79	39(49.9)	18.52	0.731, 61.486	6.424	0.683,60.415
**Visual status**						
Normal vision	115	21(18.3)	1.00		1	
Loss of vision	115	57(49.6)	4.399	2.419, 7.998	4.559	2.161,9.617

Abbreviations: COR, Crude Odd Ratio; AOR, Adjusted Odd Ratio

Of the patients with visual loss, 79.1% lost vision only in one eye and the remaining 20.9% lost vision in both eyes. Patients who lost vision in both eyes had a higher rate of psychological distress (75%) than those who lost vision in one eye only (42.9%) (Table 3). The causes of irreversible visual loss identified in this study were glaucoma (42.6%), trauma (14.8%), retinal detachment (13%) and corneal opacity (11.3%). In bivariate analysis, the level of visual acuity in the better eye and laterality of visual loss were significantly associated with psychological distress. In multivariate analysis where laterality of visual loss, pattern of visual loss, cause and duration of visual loss were entered as covariates, the probability of having psychological distress was 4 fold higher in those with bilateral vision loss than those with unilateral vision loss (AOR 4; 95% CI 1.453-11.015). Similarly, the probability of having psychological distress was 4 fold higher among patients with visual acuity of less than 3/60 in the better eye compared to those patients with visual acuity better than 3/60 (AOR 3.66; 95% CI 1.27-10.54) (Table 3). In the adjusted analysis, the cause of visual loss (AOR 0.83; 95% CI 0.186-3.711), pattern of visual loss (AOR 1.14; 95% CI 0.297- 4.372), duration of visual loss (AOR 1.106; 95% CI 0.425-2.878) and demographic variables (marital status, level of education, type of occupation, family income and living arrangement) did not influence the likelihood of having psychological distress (Table 2 & 3).

**Table 3:** Correlation between psychological distress and clinical characteristics of visual loss.

**Variables**	**Loss of vision (n=115)**	**Psychological distress**	**AOR**	**95% CI**	**P-value**
	**No (%)**	**No (%)**			
**Visual acuity in the better eye**					
>3/60	91 (79.1)	39(42.9)	1		
<3/60	24(20.9)	18 (75.0)	3.66	1.27,10.54	0.016
**Laterality of visual loss**					
One eye	91(79.1)	39(42.9)	1		
Both eyes	24(20.9)	18(75.0)	4.00	1.453,11.015	0.007
**Pattern of visual loss**					
Progressive	93(80.9)	57(50.5)	1		
Sudden	22(19.1)	10(45.5)	1.14	0.297,4.372	0.849
**Duration of visual loss**					
< 1 year	30(26.1)	14(46.6)	1		
> 1year	85(73.9)	43(50.6)	1.106	0.425,2.878	0.836
**Causes of visual loss**					
Glaucoma	49(42.6)	23(46.9)	1		
Trauma	17(14.8)	7(42.2)	0.83	0.186, 3.711	0.807
Corneal opacity	13(11.3)	6(46.2)	0.908	0.249, 3.316	0.884
Retinal detachment	15(13.0)	7(46.70	1.047	0.311, 3.524	0.941
Uveitis	10(8.7)	6(60)	1.541	0.360, 6.596	0.560
Others	11(9.6)	8(72.7)	2.359	0.526, 0.569	0.262

Abbreviations: COR, Crude Odd Ratio; AOR, Adjusted Odd Ratio

## Discussion

In this study, we found a high (33.9%) overall prevalence of psychological distress which was consistent with the findings of other similar study done on non-ophthalmic patients but using the same instrument in Zenebework-Alert Hospital, Addis Ababa which reported 34.6% [17]. However, other hospital-based studies in the country reported a lower prevalence of psychological distress ranging from 6.8 % to 18.0 % [21-23].

Our study showed a significantly higher (49.6 %) prevalence of psychological distress in patients with visual loss as compared to patients with normal vision (18.4 %). This might be due to loss of visual function for social communication, written communication, and various independent living skills including mobility, which variously results in a loss of personal independence [3]. Rural community based studies in Ethiopia that used a similar cut-off point as our study reported a 17 % prevalence of psychological distress [14,19], which is comparable to the prevalence in our control group. The higher prevalence in patients with visual loss in our study might reflect the particular psychosocial stressors experienced by patients with loss of vision. 

Though the study data collection instruments are different, the prevalence of psychological distress in patients with visual loss at least in one eye (49.6%) is comparable to the prevalence of psychological distress reported in leprosy patients (52.4%) [17] and patients with chronic obstructive pulmonary disease (COPD) (49%) [24]; but higher than the prevalence of psychological distress in patients with HIV (30.3%) [25], heart failure (32.5%) [26], cancer (35.1%) [27], Type I diabetes (35.5%) [28] and renal failure on hemodialysis (31%) [29]. This demonstrates that blindness or loss of vision is perceived by most people as paradigm of disaster of high concern as compared to other illnesses.

Psychological distress was significantly associated with level of visual acuity in the better eye. It seems that as level of visual acuity in the better eye gets worse the risk of psychological distress increases. Similarly, visual loss in both eyes increases the risk of psychological distress. This indicates that patients with severe vision loss are more prone to develop psychosocial distress as vision is central to the way one lives. The higher rate of psychological distress in patients with severe visual loss might significantly reduce the health related quality of life of the patient and add to the disability of affected persons [3]. Hence, secondary prevention of psychological distress in patients with visual loss is beneficial. However, further studies are needed to explain the mechanisms how loss of vision causes psychological morbidity. 

In our study, cause of visual loss, pattern of visual loss and duration of visual loss was not associated with psychological distress. This effect of vision loss on psychological distress appeared to be directly related to the severity of visual impairment but not to the underlying eye condition [30]. Our findings suggest that recognition and treatment of psychological morbidity should be incorporated to rehabilitation programs for persons with loss of vision. Specific psychosocial interventions might be developed for those most at risk.

Though this study is the first of its kind in the region to provide data about the association of vision loss and psychological distress among patients with visual loss in comparison with age- and sex-matched controls, it has its own limitations. The study instruments allowed identification of non-specific psychological distress only. Moreover, the cross-sectional nature of our study design limits establishing causal relationship between visual loss and psychological distress. 

In conclusion, our study showed that prevalence of psychological distress is significantly higher in patients with loss of vision compared to patients with normal vision. The effect of vision loss on psychological distress appeared to be directly related to the severity of visual impairment but not to the underlying eye conditions. Psychosocial care should be integrated to the current medical and surgical care of patients with vision loss. Future studies to characterize the nature and severity of mental disorders in this group are recommended.
